# Data-Driven Discovery of Novel Biology: From Genetics and Omics to Therapeutic Targets for Vascular Disease

**DOI:** 10.1016/j.jacbts.2025.101394

**Published:** 2025-10-27

**Authors:** Marios K. Georgakis

**Affiliations:** Institute for Stroke and Dementia Research (ISD), LMU University Hospital, LMU Munich, Munich, Germany; Program in Medical and Population Genetics, Broad Institute of MIT and Harvard, Cambridge, Massachusetts, USA

**Keywords:** coronary heart disease, genomics, monocytes, omics, proteomics

**Figure:**
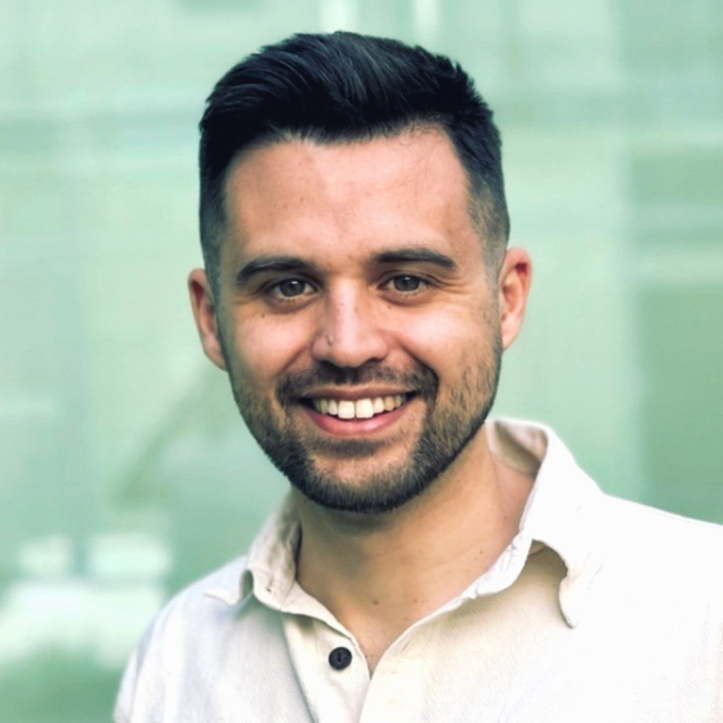

Historically, the discovery of actionable disease-causing mechanisms was largely hypothesis-driven. This paradigm is shifting. Sequencing of the human genome and, more recently, advances in deep molecular profiling now enable systematic, hypothesis-free assessment of mechanisms with therapeutic potential, a transformation increasingly shaping cardiovascular drug development.^1^ Hypothesis-free testing through genome-wide association studies has uncovered thousands of risk-driving variants for human disease, including hundreds implicated in cardiovascular pathologies. Although genetic support for a therapeutic target significantly increases the chances for successful drug development,^2^ the challenge lies in moving from variants to mechanisms. Many variants fall within noncoding regions or reside within gene-dense coding regions, where high correlation with nearby variants caused by linkage disequilibrium complicates pinpointing the causal gene. Deep molecular phenotyping with transcriptomics, proteomics, metabolomics, and other omics layers offers an alternative way for uncovering potentially actionable mechanisms at scale. Just as genetics provided a first generation of hypothesis-free discoveries, the next wave may emerge from integrating genetics with downstream omics, bridging these layers to human phenotypes.

In a new study in this issue of *JACC: Back to Translational Science*, Tahir et al^3^ used plasma proteomics as a starting point for target discovery in 1,967 self-identified Black participants from the population-based Jackson Health Study. Proteins were quantified at baseline with the aptamer-based SomaLogic 1.3K platform and were related to incident coronary heart disease (CHD) events over 13.5 years of follow-up. After correction for multiple testing, 91 proteins were associated with incident CHD in age-, sex-, and batch-adjusted analyses, and 16 remained significant in multivariable models. Many known cardiovascular risk biomarkers emerged, including N-terminal pro–B-type natriuretic peptide, GDF-15, and cystatin C. Notably, circulating SECTM1 was among the proteins significantly associated with incident CHD risk (HR per SD: 1.35; 95% CI: 1.2-1.5; FDR Q value = 0.004; top vs bottom quartile HR: 2.21; 95% CI: 1.54-3.18). This observation was externally replicated in the Cardiovascular Health Study, a predominantly White, older cohort. This potentially novel signal drove further mechanistic exploration.

Specifically, the authors combined population genetics with in vitro analyses. Building on prior evidence suggesting SECTM1 to act as a monocyte chemoattractant, they found SECTM1 levels to be associated with circulating monocyte counts in the Jackson Heart Study. Subsequently, they performed a genome-wide association study for SECTM1 levels and identified a *cis*-variant within an enhancer of *SECTM1* (rs116473040). In a Mendelian randomization framework, they found that the allele increasing plasma SECTM1 levels is also associated with monocyte percentage of white blood cells in large-scale publicly available data set, including a study of 430,000 individuals from the Million Veterans Program. Although SECTM1 levels might be confounded by other metrics, the germline genetic variant cannot, thus suggesting a causal involvement of SECTM1 in determining circulating monocyte counts. Diving deeper into these insights, the authors pursued in vivo experiments in mice demonstrating that administration of recombinant SECTM1a also led to increases in circulating monocytes, driven by the proinflammatory Ly6C^hi^ subset, known to be the ones accumulating in preclinical atherosclerotic lesions.^4^

Monocyte recruitment from the bone marrow and the circulation to atherosclerotic lesions has long been implicated as a causal driver of cardiovascular risk. Proinflammatory monocytes (Ly6C^hi^ in mice and CD14+ CD16− in humans) are a key source of plaque macrophages that drive local inflammation, promoting plaque vulnerability and destabilization. Prior work has highlighted monocyte chemoattractants as central regulators of atheroprogression. Most notably, the CCL2/CCR2 axis, which governs proinflammatory monocyte trafficking to atherosclerotic plaques, is supported by animal models, human plaque histology, epidemiological studies, and human genetics.^5^ Other chemokines with varying levels of evidence as monocyte chemoattractors, including CCL5, CX3CL1, CCL7, and CCL8, have also been implicated in atherosclerosis.^6^ SECTM1, although less studied, is now added to this list as another potential regulator of monocyte recruitment to lesions.

Despite strong evidence of causality, these pathways have not yet been tested in phase 3 clinical trials. Among anti-inflammatory treatments tested in recent years, only colchicine, a broad-spectrum agent, has been approved for lowering cardiovascular risk in patients with atherosclerosis.^7^ Several therapies are now in late-stage development, including 3 antibodies targeting the central proinflammatory cytokine interleukin-6. As strategies to reduce cardiovascular risk by targeting inflammation mature, more atherosclerosis-specific immunotherapies are likely to enter clinical testing. Discovering novel mechanisms that regulate monocyte recruitment will be key to advancing this approach.

It should be noted that although the study provides robust evidence for the involvement of SECTM1 as a regulator of monocyte counts in circulation, it does not establish a causal role in atherosclerotic cardiovascular disease. Observational associations are often confounded or subject to reverse causation, and Mendelian randomization is a valuable tool to mitigate these biases. Although the authors demonstrate that genetically predicted SECTM1 levels, proxied by a sentinel *cis*-variant, are associated with monocyte count—supporting a causal role in leukocyte biology—these effects did not extend to CHD (Figure 1). This null association may reflect limited statistical power of a weak genetic instrument, but it highlights the need for replication in larger studies. Additional limitations include the attenuation of the SECTM1 signal in the external Cardiovascular Health Study and the short-term design of the in vivo experiments, which did not assess effects on atherosclerotic lesions. Finally, high-throughput proteomic measurements are known to be platform-sensitive: quantification of the same protein often varies across aptamer-, antibody-, and mass spectrometry–based assays.^8^ Cross-platform validation, which was not performed here, remains critical.

**Figure 1:**
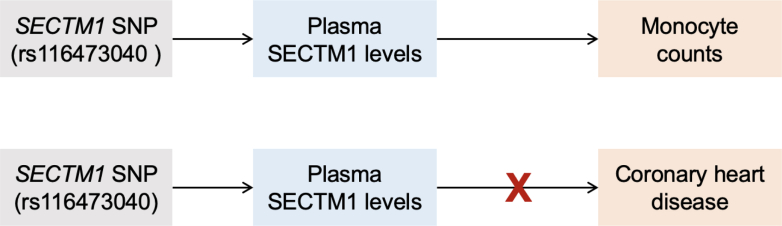
Associations of Genetically Predicted SECTM1 Levels Proxied by a SNP in the Enhancer of SECTM1 With Monocyte Counts and Coronary Artery Disease Risk

Despite these limitations, the study follows a hypothesis-free framework for biological discovery that we are likely to increasingly see in the future. Whether SECTM1 proves to be a relevant therapeutic target remains to be seen. But, as technology for molecular profiling continues to broaden, including single-cell and spatial assessments, integrating multi-omics with careful biological validation promises to yield the next generation of drug targets in cardiovascular medicine.

## Funding Support and Author Disclosures

The author has reported that he has no relationships relevant to the contents of this paper to disclose.
